# SMAD6, positively regulated by the DNM3OS-miR-134-5p axis, confers promoting effects to cell proliferation, migration and EMT process in retinoblastoma

**DOI:** 10.1186/s12935-020-1103-8

**Published:** 2020-01-22

**Authors:** Hui Wang, Xiang Ji

**Affiliations:** Ophthalmology, The People’s Hospital of Jiaozuo City, 267 Jiefang Middle Road, Jiaozuo City, 454150 Henan Province China

**Keywords:** SMAD6, DNM3OS, miR-134-5p, Retinoblastoma

## Abstract

**Background:**

Retinoblastoma (RB) is acknowledged to be the commonest intraocular malignancy in infants and children and the outcome of RB patients is unfavorable due to limited early diagnosis and effective therapy. SMAD family member 6 (SMAD6) has been reported in the initiation and progression of human cancers by acting as a biological participant. However, the role of SMAD6 in RB has not been illustrated yet.

**Methods:**

The expression of SMAD6 mRNA, miR-134-5p and DNM3OS was measured by RT-qPCR. SMAD6 protein levels were measured by western blot. The effects of SMAD6 depletion on RB cells were analyzed using CCK-8 and transwell assays. The key proteins related to epithelial-mesenchymal transition (EMT) was determined by western blot. The localization of DNM3OS was detected by nuclear/cytoplasmic assay. In addition, the direct interaction between miR-134-5p and SMAD6 or DNM3OS was confirmed with the application of dual-luciferase reporter assay.

**Results:**

SMAD6 was upregulated in RB tissue samples and cell lines, and silencing SMAD6 suppressed cell proliferation, migration and EMT in RB. Mechanically, SMAD6 was positively regulated by lncRNA DNM3OS through competitively interacting with miR-134-5p. DNM3OS contributed to RB progression by SMAD6-mediated manner.

**Conclusions:**

This research unmasked a novel DNM3OS/miR-134-5p/SMAD6 pathway in RB, which might make contribution to treatment of RB.
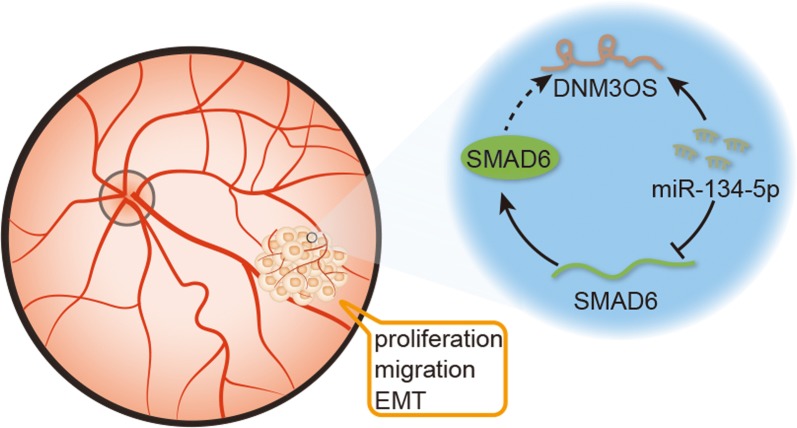

## Background

Retinoblastoma (RB) is known as the most common intraocular malignancy in infants and children [1]. There is a RB case diagnosed in every 15,000 to 20,000 neonates all over the world [2]. Among all human cancer types, RB accounts for approximately three percent. RB patients, especially those in the developing countries, could not be diagnosed in the early stage or receive effective treatment and thus present to have unfavorable prognosis [3]. Consequently, RB garnered much attention of clinicians [4].

SMAD family member 6 (SMAD6) is located in Chromosome 15. Intracellular SMAD proteins play a pivotal role in transcriptional activation of downstream genes synergistically with transcription factors via mediating the canonical TGF-β signaling pathway. SMAD6 has been reported to participate in initiation and progression of many human cancers. For example, SMAD6 is a factor for the poor survival in patients with NSCLC and its silencing contributes to the re-establishment of TGF-β homeostasis [5]. SMAD6 recovers the growth inhibition induced by TGF-beta in COLO-357 pancreatic cancer cells [6]. SMAD6 determines invasive behavior of breast cancer cells in BMP-regulated zebrafish xenograft model [7]. Nevertheless, the function of SMAD6 in RB has not been investigated yet.

In this study, we planned to prove the role of SMAD6 in RB progression and its related molecular mechanism.

## Materials and methods

### Tissue samples

30 primary RB tissues were obtained from patients with RB who were diagnosed at the People’s Hospital of Jiaozuo City. 30 normal retina tissues used as controls were collected from the People’s Hospital of Jiaozuo City. The subjects have not been subjected to any biotherapy or chemotherapy treatment prior to recruitment to this study. Informed consent was obtained and the Ethics Committee of the People’s Hospital of Jiaozuo City approved this study.

### Cell lines and cell culture

Human RB cell lines (WERI-Rb1, Y-79, HXO-RB44, and SO-Rb50), and the noncancerous human retinal pigment epithelial ARPE-19 cells were provided by the American Type Culture Collection (ATCC, VA, USA). Cells were all preserved in an incubator added with RPMI-1640 medium including 10% fetal bovine serum (FBS), 100 μg/ml streptomycin, 100 IU/ml penicillin, and 20 mM glutamine. The incubator was humidified and contained 5% CO_2_. The temperature was kept at 37 °C.

### Cell transfection

The short hairpin RNAs (shRNAs) targeting SMAD6 or DNM3OS were synthesized by GenePharma (Shanghai, China). The miR-134-5p mimics and NC-mimics were also manufactured by GenePharma. The pcDNA3.1 vectors for overexpression of SMAD6 were provided by GeneChem (Shanghai, China). The aforementioned plasmids were introduced into Weri-Rb1 andY79 cells using lipofectamine 3000 reagent (Invitrogen).

### RNA extraction and quantitative real-time PCR (RT-qPCR)

Extraction of the total RNA from cells or tissues was made by using RNAiso Plus (Takara, Japan) and next reversely transcribed into cDNAs using a Taqman Advanced miRNA cDNA Synthesis Kit (MA, USA) and a Reverse Transcript Kit (Applied Biosystems, CA). Quantitative real-time PCR was conducted using a FastStart Universal SYBR Green Master Kit (Roche, Basel, Switzerland). U6 and GAPDH were used for the normalization of miRNA or lncRNA, respectively. The sequences of the primers were: GAPDH, forward, 5′-GGGAGCCAAAAGGGTCAT-3′ and reverse, 5′-GAGTCCTTCCACGATACCAA-3′; U6, forward 5′-GCTTCGGCAGCACATATACTAAAAT-3′ and reverse: 5′-CGCTTCACGAATTTGCGTGTCAT-3′; SMAD6, forward 5′-TTGCAACCCCTACCACTTCA-3′ and reverse 5′-TTGGTGGCATCTGGAGACAT-3′; miR-134-5p, forward, 5′-ACACTGCATCCTGGCAATTC-3′ and reverse, 5′-CGTGGTGAATCGAGACTCAC-3′; DNM3OS, forward, 5′-GGTCCTAAATTCATTGCCAGTTC-3′ and reverse, 5′-ACTCAAGGGCTGTGATTTCC-3′.

### Cell counting kit-8 (CCK-8) assay

A CCK-8 kit (Dojindo, Japan) was utilized for analysis of cell proliferation. In brief, cells were placed into 96-well plates for 1, 2, 3, and 4 days of incubation. Next, each well was added with 10 μl of CCK-8 solution. The absorbance value (OD) was measured at the wavelength of 450 nm.

### Transwell assay

In the transwell assay, cells were placed in a 24-well plate which contained pores with 8.0 μm pores. 10% FBS medium was supplemented in the lower chamber as chemoattractant. 24 h after incubation, cells migrated to the lower surface were stained by Giemsa and counted under a microscope.

### Western blot

Separation of total protein was performed using 12% SDS polyacrylamide gel electrophoresis. The isolated proteins were then preserved on nitrocellulose membranes (Millipore, MA, USA) which were next blocked with 5% skim milk. The membranes were subjected to the incubation of primary antibodies, including SMAD6 (ab80049, Abcam), E-cadherin (ab40772, Abcam), N-cadherin (ab18203, Abcam), Vimentin (ab92547, Abcam) and GAPDH (ab8245, Abcam). Subsequently, the membranes were incubated with secondary antibody (Sigma). The proteins were assessed utilizing enhanced chemiluminescence reagents (Pierce, IL, USA).

### Dual‐luciferase reporter assay

WERI-Rb1 and Y79 cells were maintained in 96‐well plates and next co‐transfected with pmirGLO‐SMAD6-WT/Mut reporter or pmirGLO‐DNM3OS-WT/Mut reporter, and miR-134-5p mimics or NC-mimic with the help of Lipofectamine 3000 reagent. 48 h later, a Dual‐GLO Luciferase Assay System Kit (Promega, WI, USA) and a Fluorescence/Multi‐Detection Microplate Reader (BioTek, VT, USA) were utilized to analyze luciferase activity.

### RNA pull-down assay

In the RNA pull-down assay, WERI-Rb1 cells were transfected with biotinylated wild type miR-134-5p (Bio-miR-134-5p-WT) or mutant type miR-134-5p (miR-134-5p-Mut) for 24 h and next incubated in cell lysate containing magnetic beads (Ambion, Life Technologies) which were pre-coated with streptavidin. The RNA complex pulled down was subjected to RT-qPCR analysis for quantification of AC004687.1, ENTPD3-AS1, DNM3OS, AC009948.3, and AC145207.5.

### Statistical analysis

Data analyses were performed with the use of SPSS 17.0 software (SPSS, Chicago, IL). Two group comparison was made with Student’s *t* test. Dara obtained from three or more independent experiments were shown as mean ± standard deviation. One-way/two-way ANOVA was applied to compare more than two groups. Expression correlation analysis was conducted using Pearson correlation test. A *P* value under 0.05 was regarded statistically significant.

## Results

### SMAD6 knockdown inhibits RB cell proliferation, migration and epithelial-mesenchymal transition (EMT)

**Fig. 1:**
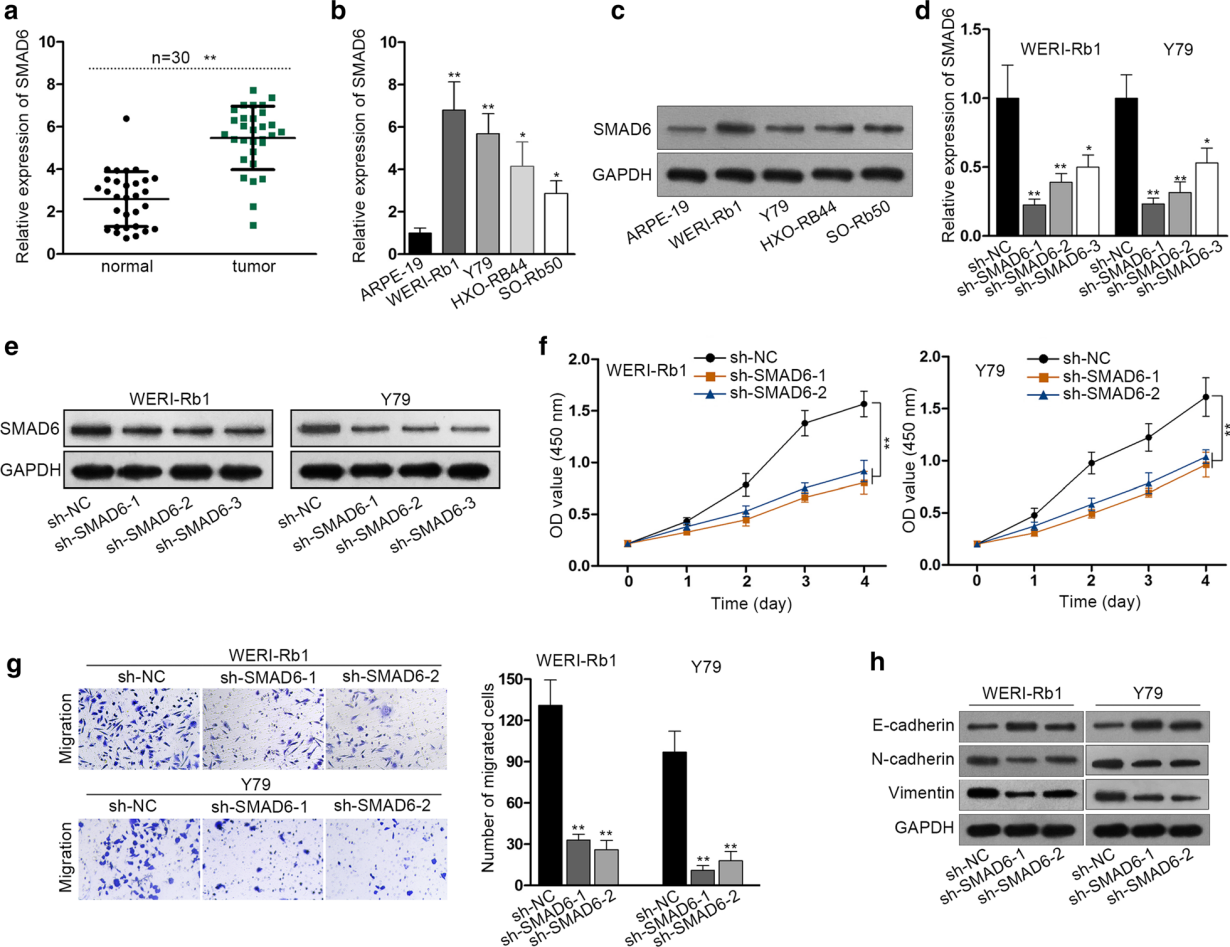
SMAD6 knockdown inhibits RB cell proliferation, migration and epithelial-mesenchymal transition (EMT). **a** RT-qPCR measured the relative expression of SMAD6 in RB and normal tissue samples. **b** RT-qPCR detected the relative expression of SMAD6 in four RB cells (WERI-Rb1, Y79, HXO-RB44, SO-Rb50) and one normal ARPE-19 cell line. **c** Western blot analyzed the protein levels of SMAD6 in four RB cell lines and the noncancerous ARPE-19 cell line. **d**, **e** sh-SMAD6-1, sh-SMAD6-2 or sh-SMAD6-3 were introduced into RB cells. RT-qPCR and western blot confirmed the knockdown efficiency of SMAD6. **f** CCK-8 assay was conducted for detection of cell proliferation. **g** Transwell assay was carried out for measurement of cell migration. **h** Western blot examined the levels of E-cadherin, N-cadherin and Vimentin. Experiments were conducted thrice. *p < 0.05, **p < 0.01

At the beginning of this study, SMAD6 level in indicated cells was measured. SMAD6 was significantly upregulated in RB tissues compared to the normal retina tissues (Fig. 1a). The expression of SMAD6 in RB cells was also examined. Not only the mRNA levels but also the protein levels of SMAD6 in RB cell lines were notably higher than those in the non-tumor cell line (Fig. 1b, c). Afterwards, the function of SMAD6 in RB cell proliferation, migration and the EMT process was evaluated. Three shRNAs targeting SMAD6 (sh-SMAD6-1, sh-SMAD6-2, sh-SMAD6-3) were introduced into WERI-Rb1 and Y79 cells for stable knockdown of SMAD6. RT-qPCR and western blot assay were used for evaluation of knockdown efficiency (Fig. 1d, e). Since the sh-SMAD6-1 and sh-SMAD6-2 groups were more efficient in reducing SMAD6 transcript, sh-SMAD6-1 and sh-SMAD6-2 were used in the subsequent loss-of-function assays. Cell proliferation was measured by CCK-8 assay and it was demonstrated that the cell proliferation was obviously decreased after SMAD6 depletion (Fig. 1f). Transwell assay was conducted for detection of cell migration and it was indicated that migration capacity of RB cells was inhibited in the presence of SMAD6 silencing (Fig. 1g). The key proteins related to EMT process (E-cadherin, N-cadherin and Vimentin) were tested by western blot, which suggested that the EMT process was retarded after SMAD6 silencing (Fig. 1h). Immunofluorescence staining indicated that the reverse effect of SMAD6 silencing on EMT process (Additional file 1: Fig. S1A). It has been reviewed that cells can metastasize in other ways except for traditional EMT [8]. For instance, cells can form clusters by detaching, occupying one or more stable hybrid epithelial/mesenchymal (E/M) phenotypes. These hybrid E/M cells, obtained both epithelial and mesenchymal markers, may possess higher potential in tumor-initiation and metastasis compared to cells in the two sides of the EMT spectrum. Our results revealed the co-existence of epithelial and mesenchymal markers, indicating the existence of hybrid E/M cells. Therefore, silenced SMAD6-mediated the decreased migration ability of RB cells may be owed to the destroy the formation of hybrid E/M cells. In this regard, we also measured the levels of various EMT-inducing factors such as ZEB1, GRHL2, OVOL2 at mRNA and/or protein levels. Silenced SMAD6 was found to be efficiently decreased the level of ZEB1 but increased the levels of GRHL2 and OVOL2 (Additional file 2: Fig. S2A, B). Furthermore, we also demonstrated that knockdown of SMAD6 in HOX-RB44 and SO-Rb50 cells led to the inhibition on cell proliferation, migration and reversed EMT process (Additional file 3: Fig. S3A–C).

### SMAD6 expression is negatively regulated by miRNA-134-5p

**Fig. 2:**
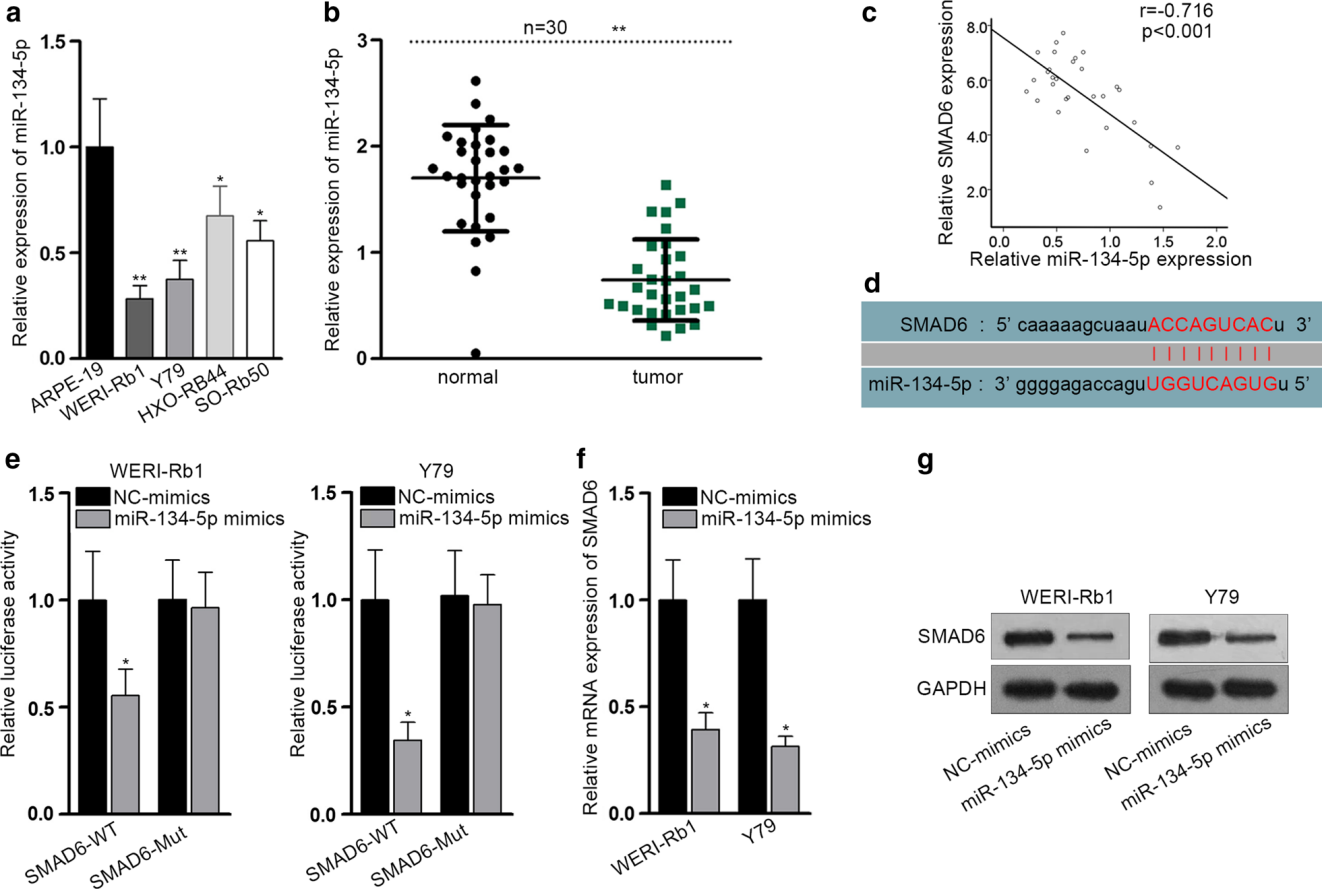
SMAD6 expression is negatively regulated by microRNA-134-5p. **a** RT-qPCR detected miR-134-5p expression in four RB cell lines by comparing to the normal cell line (ARPE-9). **b** RT-qPCR examined miR-134-5p expression in RB and normal tissue samples. **c** Pearson correlation test about the expression correlation between miR-134-5p and SMAD6. **d** Predicted binding site between SMAD6 and miR-134-5p obtained from the starBase v3.0 website. **e** Relative luciferase activity of SMAD6-WT and SMAD6-Mut was measured in indicated cells with luciferase reporter assay. **f** RT-qPCR measured SMAD6 mRNA levels after upregulation of miR-134-5p in indicated RB cells. **g** Western blot tested SMAD6 protein levels after transfection of miR-134-5p mimics. Experiments were conducted thrice. *p < 0.05, **p < 0.01

As known, mRNAs could be modulated by microRNAs (miRNAs). To search for the upstream miRNAs of SMAD6, we utilized the starBase v3.0 website (http://starbase.sysu.edu.cn/). Bioinformatics from six diagrams (PITA, miRmap, MicroT, miRanda, PicTar and TargetScan) together predicted that only miR-134-5p held the potential to be bound to SMAD6. The expression of miR-134-5p in RB cells was assessed and it was apparent that miR-134-5p was downregulated in RB cells (Fig. 2a). Concordantly, the expression of miR-134-5p was decreased in RB tissues (Fig. 2b). Conducting Pearson correlation test, we found the negative correlation between miR-134-5p and SMAD6 expression (Fig. 2c). The binding sequence in miR-134-5p with SMAD6 was listed in Fig. 2d. Luciferase activity assay demonstrated that the relative luciferase activity of SMAD6-WT was strikingly suppressed by miR-134-5p overexpression (Fig. 2e). In addition, RT-qPCR and western blot indicated that miR-134-5p overexpression resulted in decreased SMAD6 mRNA and protein levels in RB cells (Fig. 2f, g). Thus, it can be summarized that SMAD6 is a target of miR-134-5p and is negatively modulated by it.

### Upregulation of miR-134-5p led to the suppression on the proliferation, migration and EMT in RB

**Fig. 3:**
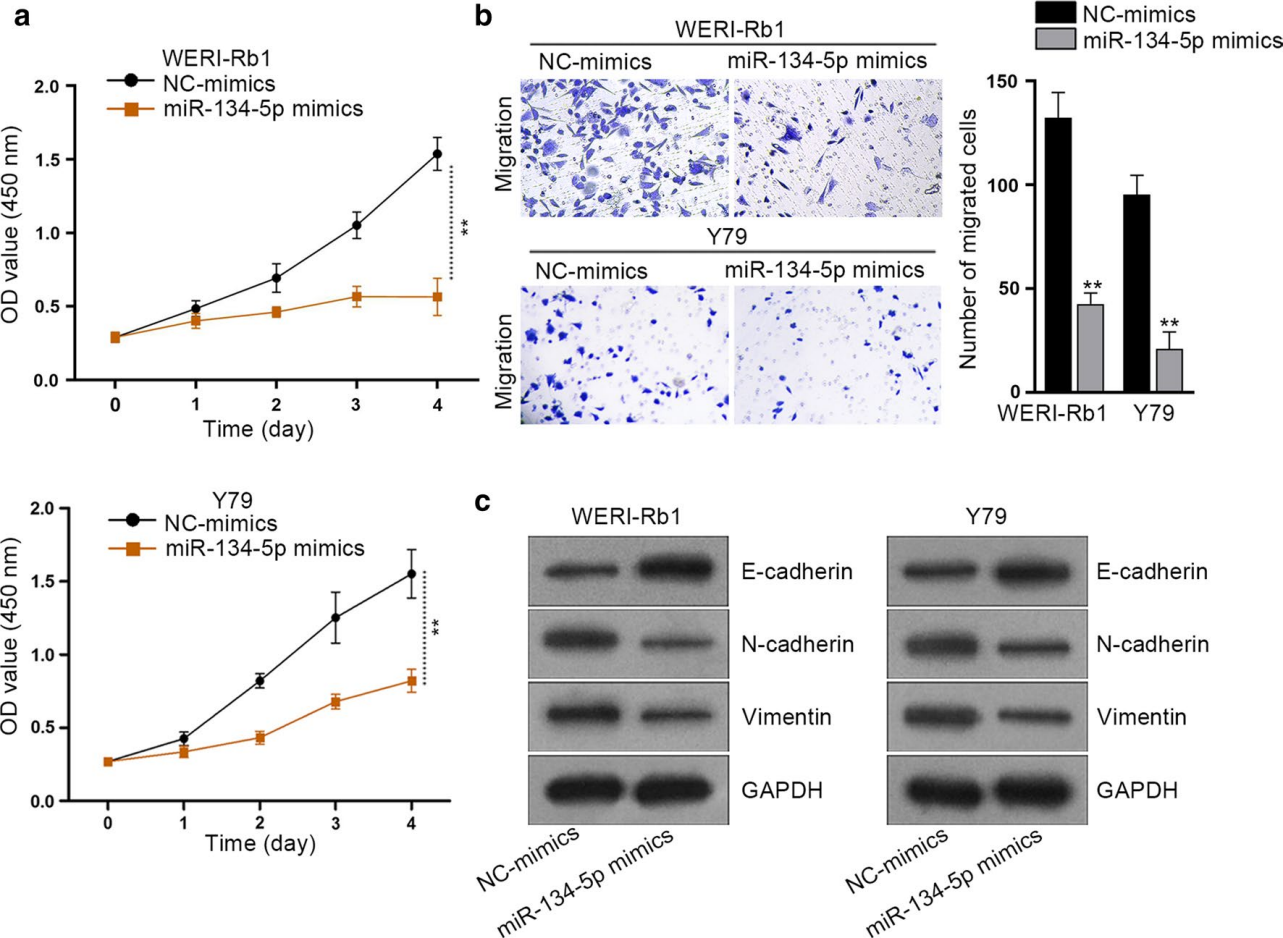
Upregulation of miR-134-5p inhibits proliferation, migration and EMT of RB cells. **a** CCK-8 assay was conducted for detection of cell proliferation after transfection of miR-134-5p mimics. **b** Transwell assay was carried out for measurement of cell migration after transfection of miR-134-5p mimics. **c** Western blot examined the levels of E-cadherin, N-cadherin and Vimentin after transfection of miR-134-5p mimics. Experiments were conducted thrice. **p < 0.01

Since the role of miR-134-5p in RB has rarely been investigated before, we wondered its precise function in the biological behaviors of RB cells. Thus, gain of miR-134-5p function assays were performed in WERI-Rb1 and Y-79 cells. As a consequence, RB cells treated with miR-134-5p mimics presented significantly lower viability than the NC-mimics group (Fig. 3a). Meanwhile, it was showed that cell migration was inhibited by ectopic expression of miR-134-5p in both the two RB cells (Fig. 3b). Moreover, the result of western blot analysis implied that the EMT process was also suppressed in RB cells in response to the treatment of miR-134-5p mimics, as E-cadherin expression was obviously enhanced while the levels of N-cadherin and Vimentin were apparently reduced in these two RB cells facing miR-134-5p upregulation (Fig. 3c). Moreover, we also applied western blot assay to determine the effect of NC mimics, miR-134-5p WT or miR-134-5p MUT on the EMT-related proteins. As depicted in Additional file 3: Figure S3D, miR-134-5p WT led to the decreased levels of SMAD6 and the reversed EMT process, mutated miR-134-5p didn’t affect the protein level of SMAD6 and EMT process.

### MiR-134-5p is sequestered from SMAD6 by DNM3OS in RB cells

**Fig. 4:**
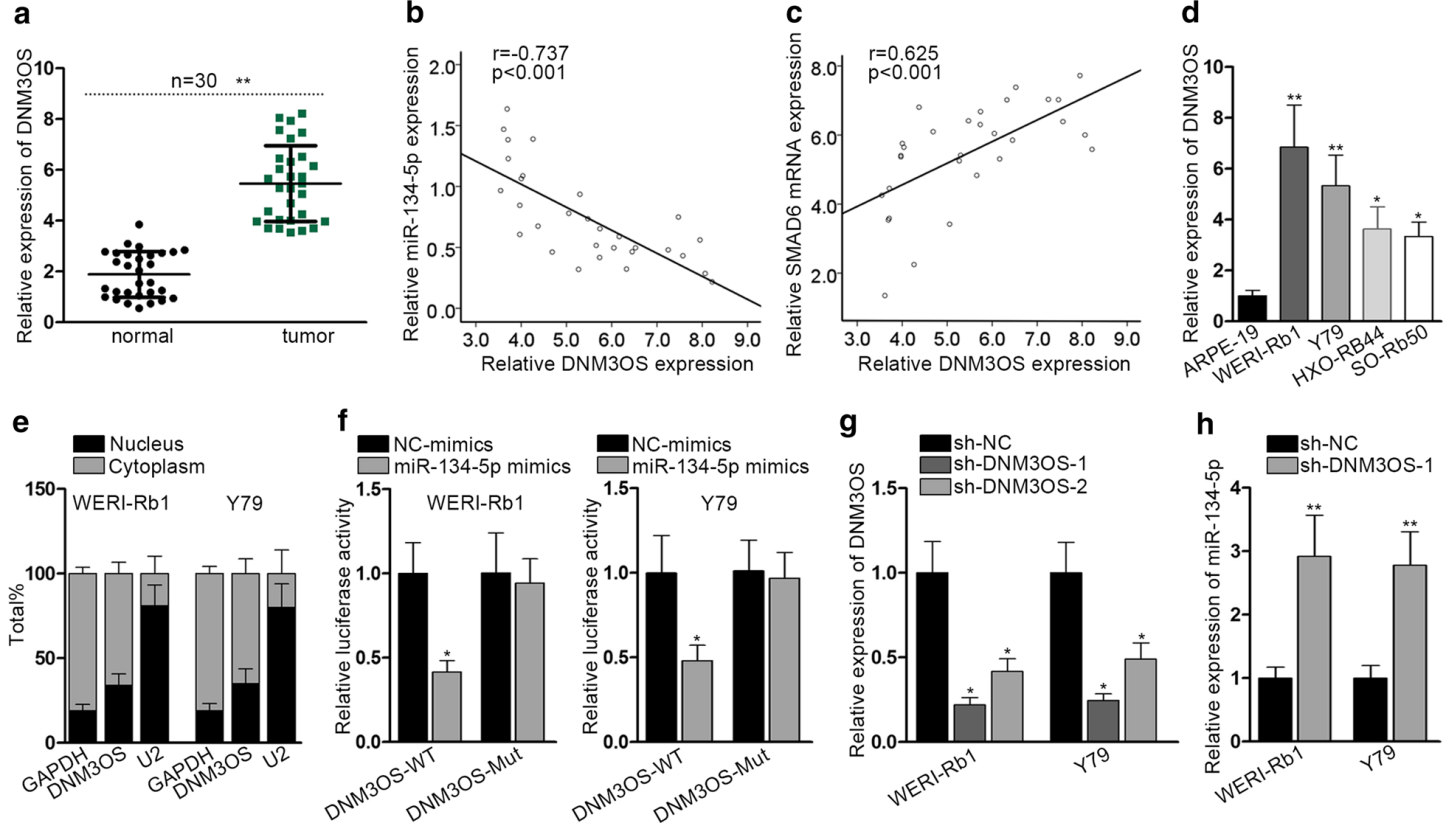
MiR-134-5p is sequestered by DNM3OS in RB cells. **a** RT-qPCR measured DNM3OS expression levels in RB and normal tissue samples. **b** Pearson correlation test analyzed the correlation of DNM3OS and miR-134-5p expression. **c** Pearson correlation test analyzed the correlation of DNM3OS and SMAD6 expression. **d** RT-qPCR measured DNM3OS expression in four RB cell lines and a normal ARPE-19 cell line. **e** Nuclear/cytoplasmic fractionation localized DNM3OS in RB cells. **f** Luciferase reporter assay evaluated the relative luciferase activity of DNM3OS-WT and DNM3OS-Mut. **g** RT-qPCR detected the knockdown efficiency of DNM3OS. **h** RT-qPCR evaluated miR-134-5p expression after DNM3OS knockdown. Experiments were conducted thrice. *p < 0.05, **p < 0.01

It has been repeatedly validated that miRNAs could be sequestered by long noncoding RNA (lncRNAs), thus we supposed that miR-134-5p was sequestered by lncRNAs in RB cells. Using starBase v3.0, we found a cluster of lncRNAs capable of binding to miR-134-5p. According to the CLIP data, AC004687.1, ENTPD3-AS1, DNM3OS, AC009948.3, and AC145207.5 were the top five candidates (Additional file 4: Fig. S4A). RNA pull-down assay indicated that DNM3OS was the one most intimately binding to miR-134-5p among the five lncRNAs (Additional file 4: Fig. S4B). Having conducted RT-qPCR, we discovered that DNM3OS was amplified in RB tissues (Fig. 4a). Additionally, Pearson correlation test demonstrated that the expression of DNM3OS and miR-134-5p in RB tissues was negatively correlated (Fig. 4b). However, the expression of DNM3OS and SMAD6 in RB tissues was positively correlated (Fig. 4c). Next, DNM3OS expression in RB cell lines was detected and we found DNM3OS was overexpressed in RB cell lines (Fig. 4d). The intracellular distribution of DNM3OS in RB cells was then examined. The results of nuclear/cytoplasmic fractionation indicated the cytoplasmic localization of DNM3OS in RB cells (Fig. 4e). Luciferase reporter assay illustrated that the luciferase activity of DNM3OS-WT was notably suppressed as a result of miR-134-5p overexpression (Fig. 4f). Thereafter, DNM3OS was silenced via introduction of sh-DNM3OS-1 and sh-DNM3OS-2 into WERI-Rb1 and Y79 cells. Sh-DNM3OS-1 was selected in the later assays considering its better knockdown efficacy (Fig. 4g). The expression of miR-134-5p was markedly increased in cells treated with sh-DNM3OS-1 (Fig. 4h). Rescue assays were carried out in WERI-Rb1 after indicated transfections. It is proved that upregulation of miR-134-5p mimics impeded the proliferation, migration and EMT process in WERI-Rb1 cells, which could be recovered by the overexpression of SMAD6 (Additional file 4: Fig. S4C–E). Moreover, we examined the regulatory effect of SMAD6 and TWIST on the expression level of DNM3OS. The results indicated that the expression level of DNM3OS was downregulated after silencing of SMAD6 and TWIST (Additional file 4: Fig. S4F), indicating that SMAD6 and TWIST may form a positive feedback loop with DNM3OS in RB. Furthermore, we also explored whether DNM3OS could directly affect the luciferase activity of SMAD6 3′UTR. No significant effect was observed (Additional file 5: Fig. S5A). Previous studies have shown that DNM3OS-derived miRNAs can regulate SMAD pathway. In this study, we also investigated whether DNM3OS-derived miRNAs could regulate SMAD6. We overexpressed these three miRNAs in two RB cells, respectively (Additional file 5: Fig. S5B). Further luciferase reporter assay indicated that upregulation of these three miRNAs had no significant effect on the luciferase activity of SMAD6 3′UTR (Additional file 5: Fig. S5C). These findings excluded the direct regulation of DNM3OS on SMAD6 and the regulatory effect of DNM3OS-derived miRNAs on SMAD6.

### DNM3OS positively regulate cell proliferation, migration and EMT of RB cells via releasing SMAD6

**Fig. 5:**
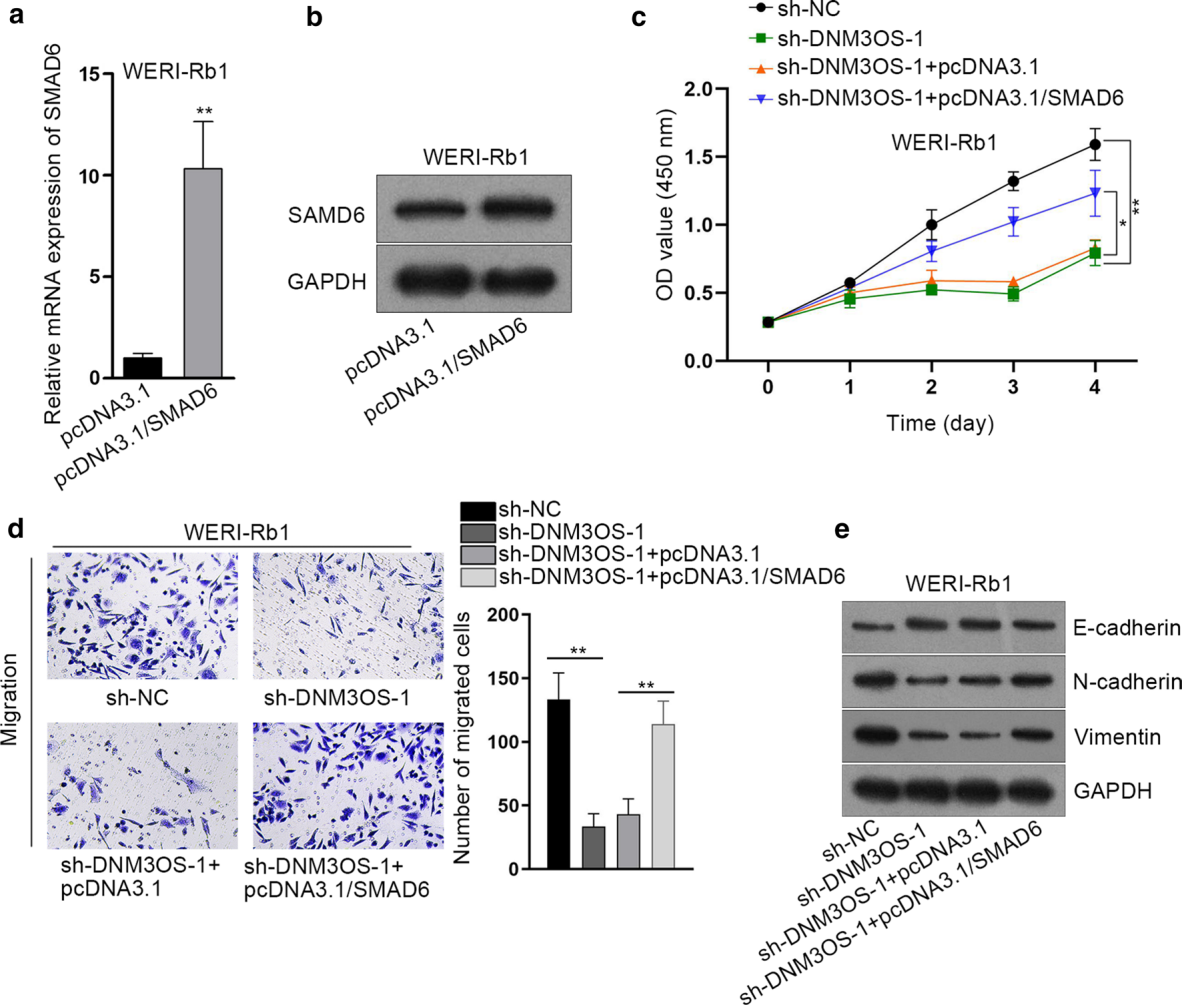
DNM3OS positively regulate cell proliferation, migration and EMT of RB cells via releasing SMAD6. **a**, **b** RT-qPCR and western blot determined the overexpression efficiency of SMAD6 in WERI-Rb1 cells. **c** CCK-8 assay was conducted for detection of cell proliferation. **d** Transwell assay was carried out for measurement of cell migration. **e** Western blot examined the levels of E-cadherin, N-cadherin and Vimentin. Experiments were conducted thrice. *p < 0.05, **p < 0.01

Rescue assays were carried out to validate whether SMAD6 was a prerequisite of DNM3OS modulate RB progression. RT-qPCR and western blot assay showed that SMAD6 was successfully overexpressed via introducing pcDNA3.1/SMAD6 into WERI-Rb1 cells (Fig. 5a, b). CCK-8 assay elucidated that silencing DNM3OS significantly inhibited RB cell proliferation, whereas upregulation of SMAD6 partly offset that effect (Fig. 5c). Likewise, the declined cell migration caused by DNM3OS silence was rescued by SMAD6 overexpression (Fig. 5d). The EMT process was retarded after DNM3OS depletion but that tendency was mitigated by SMAD6 overexpression (Fig. 5e). The above results indicated that DNM3OS requires SMAD6 to promote proliferation, migration and EMT of RB cells.

## Discussion

RB is known as a malignancy featuring with high aggressiveness. There are approximately 1000 new RB cases diagnosed in China each year [9]. About 5% of blindness in infants and children could be attributed to RB [10]. Thus, it is of great importance to explore the mechanism responsible for retinoblastoma initiation and development.

SMAD6 has been unmasked as an important regulator in a variety of human cancers [5–7, 11, 12]. Herein, we discovered that SMAD6 was upregulated in RB tissues and cells. Moreover, knockdown of SMAD6 dramatically suppressed the proliferation of RB cells.

It is common for retinoblastoma to extend into the brain along the optic nerves and distal metastasis could easily take place. During tumor metastasis, epithelial cells will pass through the extracellular matrix (ECM), transform into mesenchymal cells characterized with migration ability and then lead to distant metastasis [13–15]. In this research, we found that depletion of SMAD6 not only inhibited cell migration but also retarded the EMT process.

MiR-134-5p has been preciously elucidated as a tumor suppressor in non-small cell lung cancer [16], cervical cancer [17], and nasopharyngeal carcinoma [18]. This research validated the binding act of miR-134-5p and SMAD6. Besides, the expression of miR-134-5p and SMAD6 was negatively correlated. The biological function of miR-134-5p in RB was also detected and we observed suppressed cell proliferation, migration and EMT in RB as a result of miR-134-5p overexpression.

LncRNAs have been certified to release tumor-related genes from the inhibitory effect of miRNAs and thus regulate tumorigenesis. For example, lncRNA HEIH promotes tumorigenesis in colorectal cancer via counteracting the effect of miR-939 on the transcriptional repression of Bcl-xL [19]. LncRNA SChLAP1 contributes to the acceleration on the proliferation and metastasis of prostate cancer via regulating miR-198-mediated MAPK1 pathway [20]. MALAT1 modulates the autophagy of retinoblastoma cell through miR-124-targated stx17 [21]. DNM3 opposite strand/antisense RNA (DNM3OS), also known as DNM3-AS1 or MIR199A2HG, is located in Chromosome 1q24.3. DNM3OS has been revealed as a potential tumor promoter in some human cancers. For example, DNM3OS contributes to EMT in ovarian cancer [22]. DNM3OS confers radioresistance in esophageal squamous carcinoma [23]. DNM3OS exerted promotive role in gastric cancer by interacting with Snail to modulate tumor progression and EMT [24]. In this study, we addressed the expression pattern and functional role of DNM3OS in RB. High level of DNM3OS was firstly uncovered in RB cells and tissue samples. The negative correlation of miR-134-5p with DNM3OS or SMAD6 was analyzed. EMT and/or MET inducing factors such as ZEB1, GRHL2, OVOL2 have been reported in a previous study [25]. EMT also has been taken as an example to illustrate how phenotypic plasticity enables cancer cells to acquire hybrid phenotypes, thus making them tend to be more aggressive [26]. In this study, we also assessed the expression level of these factors in response to SMAD6 knockdown. The protein level of ZEB1 was decreased but the levels of GRHL2 and OVOL2 were increased in SMAD6-downregulated RB cells. Eventually, rescue assays illustrated that DNM3OS depletion made a dent in proliferation, migration and EMT of RB cells, while that tendency was offset by SMAD6 overexpression. DNM3OS has been reported to be transcriptionally activated by TWIST [22]. Meanwhile, the expression level of DNM3OS was measured in SMAD6-silenced RB cells. The positive regulatory effect was observed. Then, we also assessed the expression level of DNM3OS in response to the downregulation of TWIST or SMAD6. The expression level of DNM3OS was significantly decreased in indicated RB cells. Therefore, SMAD6 and DNM3OS may form a positive feedback loop, thus help to find novel molecular pathway in regulating EMT process in RB.

## Conclusion

This research unmasked a novel DNM3OS/miR-134-5p/SMAD6 pathway in RB and we believe our findings will make contribution to treatment of RB.

## Supplementary information

**Additional file 1: Figure S1.** (A) Immunofluorescence staining was used to assess the intensity of E-cadherin and N-cadherin in two RB cells transfected with sh-NC, sh-SMAD6-1 or sh-SMAD6-2. Experiments were conducted thrice. Experiments were conducted thrice.

**Additional file 2: Figure S2.** (A, B) mRNA and protein levels of ZEB1, GRHL2 and OVOL2 were evaluated in SMAD6-downregulated RB cells. Experiments were conducted thrice. **p < 0.01.

**Additional file 3: Figure S3.** (A) CCK-8 assay was applied to measure cell proliferation in HXO-RB44 and SO-Rb50 cells after silencing of SMAD6. (B) Transwell migration assay in RB cells transfected with SMAD6-specific shRNAs or sh-NC. (C) EMT markers IN SMAD6-downregulated RB cells. (D) SMAD6 and EMT markers were detected in RB cells transfected with NC mimics, miR-134-5p-WT, miR-134-5p-MUT. **p < 0.01.

**Additional file 4: Figure S4.** (A) The binding sites of AC004687.1, ENTPD3-AS1, DNM3OS, AC009948.3, or AC145207.5 and miR-134-5p were predicted and obtained from starBase v3.0. (B) RNA pull-down assay followed by RT-qPCR was carried out to prove the interaction between lncRNAs and miR-134-5p. (C–E) Cell proliferation, migration and EMT process were detected in RB cells after required transfection. (F) The DNM3OS expression was measured in cells transfected with sh-NC, sh-SMAD6 or sh-TWIST. Experiments were conducted thrice. **p < 0.01, ***p < 0.001.

**Additional file 5: Figure S5.** (A) Luciferase activity of vectors containing SMAD6 3′UTR was measured in RB cells in response to the silencing of DNM3OS. (B) Overexpression of miR-199-3p, miR-199a-5p and miR-214-3p in two RB cells by indicated miRNA mimics. (C) Luciferase activity of vectors containing SMAD6 3’UTR was measured in RB cells after inducing the upregulation of three miRNAs. **p < 0.01; NS: no significance.

## Data Availability

Not applicable.

## Abbreviations

ANOVA: analysis of variance
DNM3OS: DNM3 opposite strand/antisense RNA
GAPDH: glyceraldehyde-3-phosphate dehydrogenase
lncRNA: long non-coding RNA
mRNA: messenger RNA
RPMI-1640: Roswell Park Memorial Institute-1640
RT-qPCR: quantitative real-time polymerase chain reaction
SDS: sodium dodecyl sulfate
SMAD6: SMAD family member 6
MiRNA: microRNA
