# Supplementary figures and images for: SMAD6, positively regulated by the DNM3OS-miR-134-5p axis, confers promoting effects to cell proliferation, migration and EMT process in retinoblastoma
Source: Cancer Cell Int. 2020 Jan 22;20:23. doi: 10.1186/s12935-020-1103-8 (PMC6977187; doi:10.1186/s12935-020-1103-8)

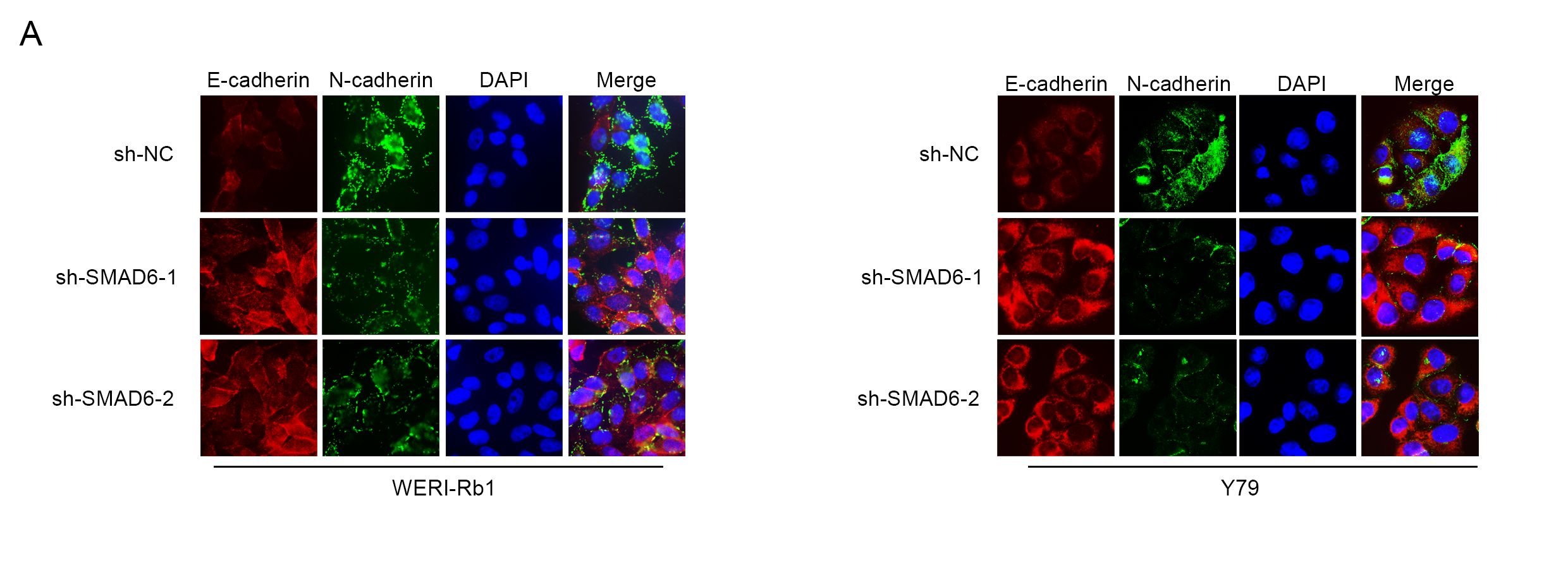

Supplement: Supplementary file 1 — Additional file 1: Figure S1. (A) Immunofluorescence staining was used to assess the intensity of E-cadherin and N-cadherin in two RB cells transfected with sh-NC, sh-SMAD6-1 or sh-SMAD6-2. Experiments were conducted thrice. Experiments were conducted thrice. [file 12935_2020_1103_MOESM1_ESM.tif]

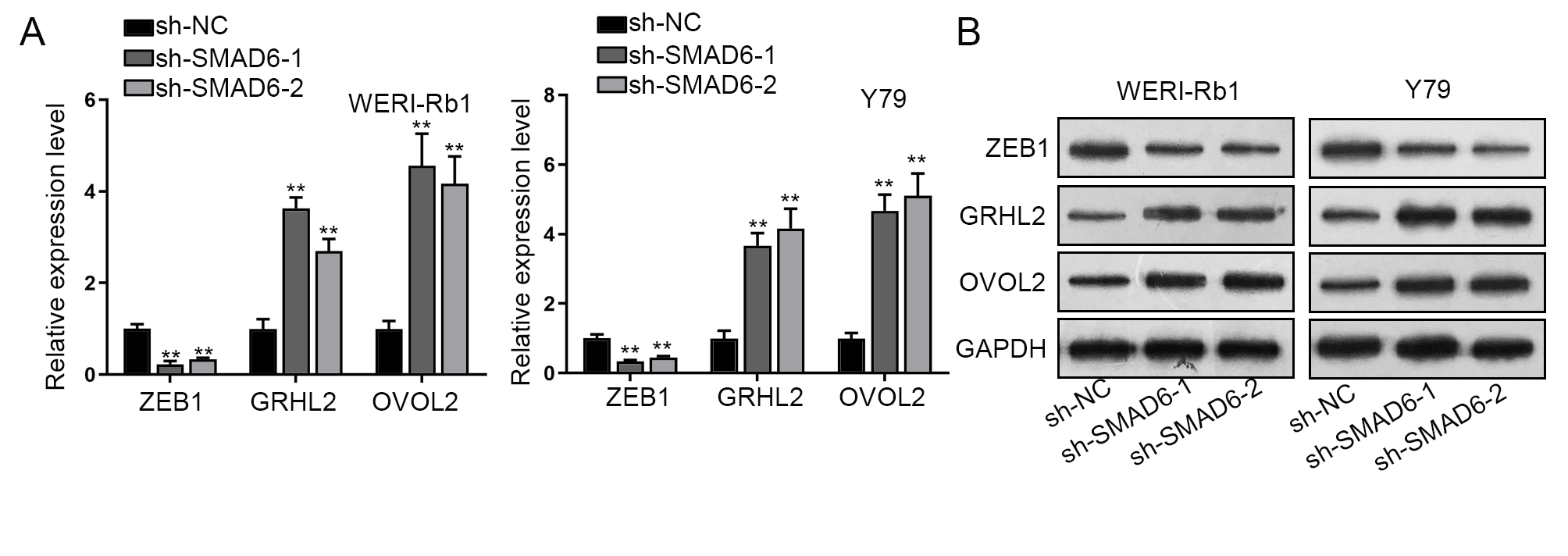

Supplement: Supplementary file 2 — Additional file 2: Figure S2. (A, B) mRNA and protein levels of ZEB1, GRHL2 and OVOL2 were evaluated in SMAD6-downregulated RB cells. Experiments were conducted thrice. **p < 0.01. [file 12935_2020_1103_MOESM2_ESM.tif]

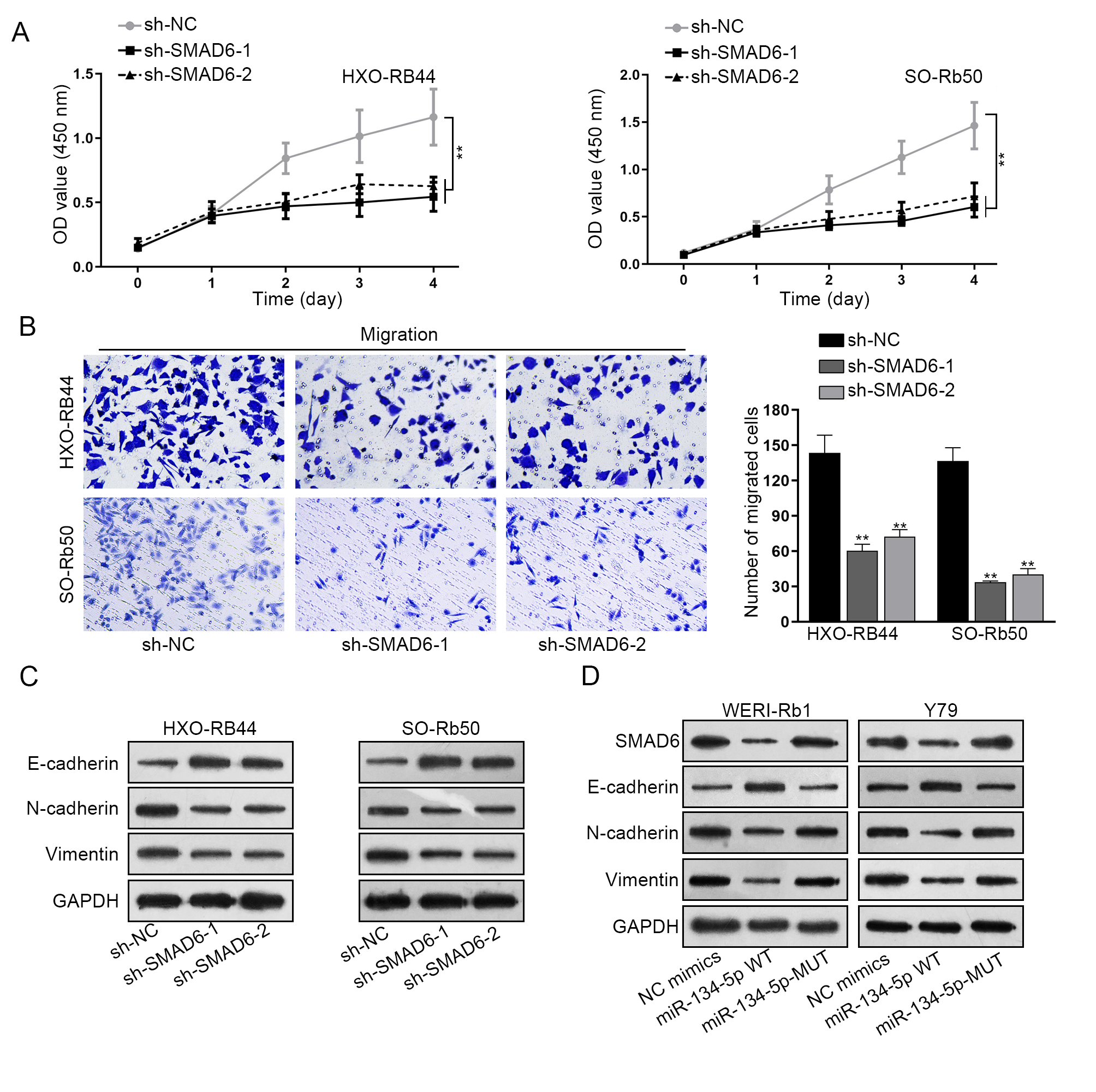

Supplement: Supplementary file 3 — Additional file 3: Figure S3. (A) CCK-8 assay was applied to measure cell proliferation in HXO-RB44 and SO-Rb50 cells after silencing of SMAD6. (B) Transwell migration assay in RB cells transfected with SMAD6-specific shRNAs or sh-NC. (C) EMT markers IN SMAD6-downregulated RB cells. (D) SMAD6 and EMT markers were detected in RB cells transfected with NC mimics, miR-134-5p-WT, miR-134-5p-MUT. **p < 0.01. [file 12935_2020_1103_MOESM3_ESM.tif]

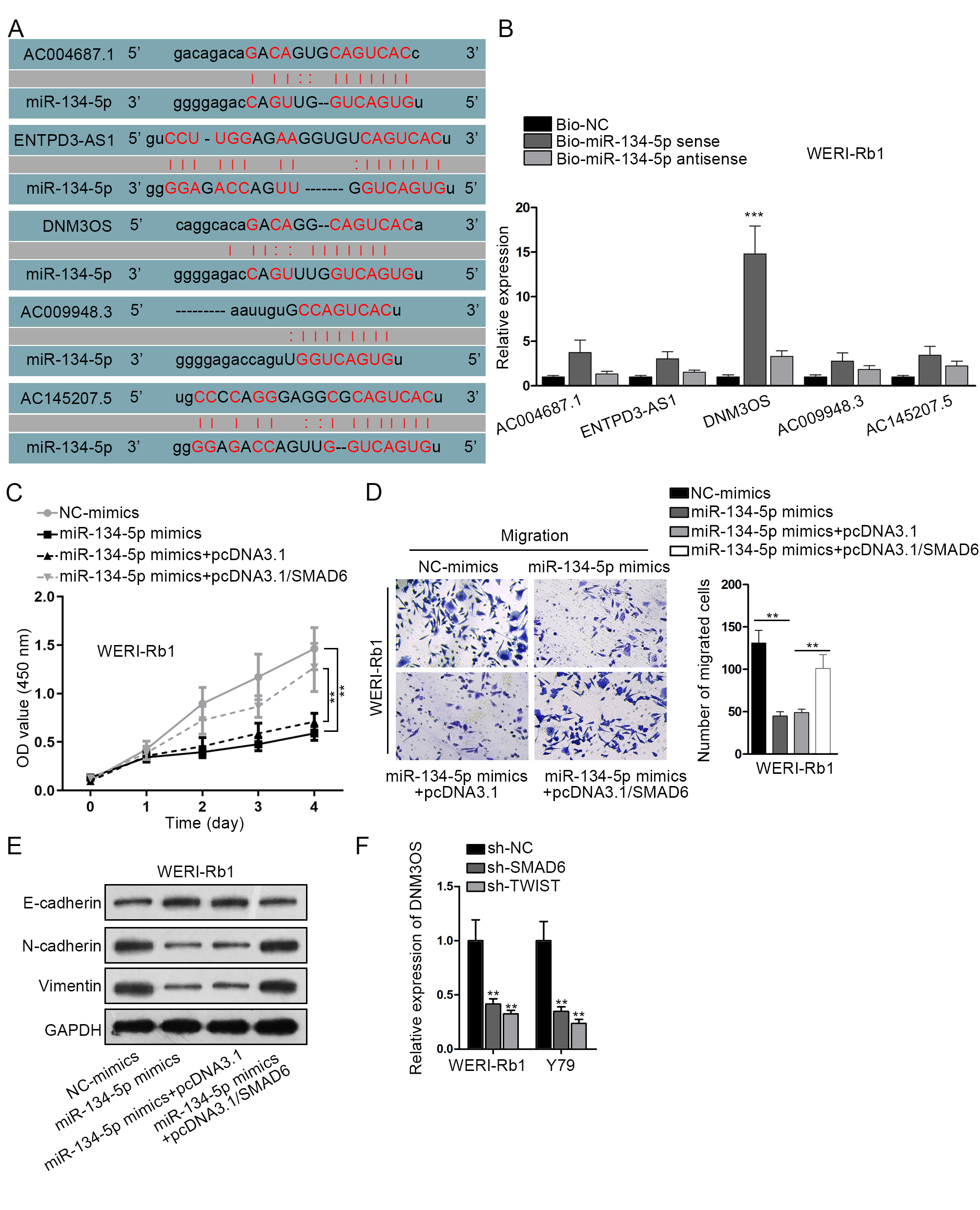

Supplement: Supplementary file 4 — Additional file 4: Figure S4. (A) The binding sites of AC004687.1, ENTPD3-AS1, DNM3OS, AC009948.3, or AC145207.5 and miR-134-5p were predicted and obtained from starBase v3.0. (B) RNA pull-down assay followed by RT-qPCR was carried out to prove the interaction between lncRNAs and miR-134-5p. (C–E) Cell proliferation, migration and EMT process were detected in RB cells after required transfection. (F) The DNM3OS expression was measured in cells transfected with sh-NC, sh-SMAD6 or sh-TWIST. Experiments were conducted thrice. **p < 0.01, ***p < 0.001. [file 12935_2020_1103_MOESM4_ESM.tif]

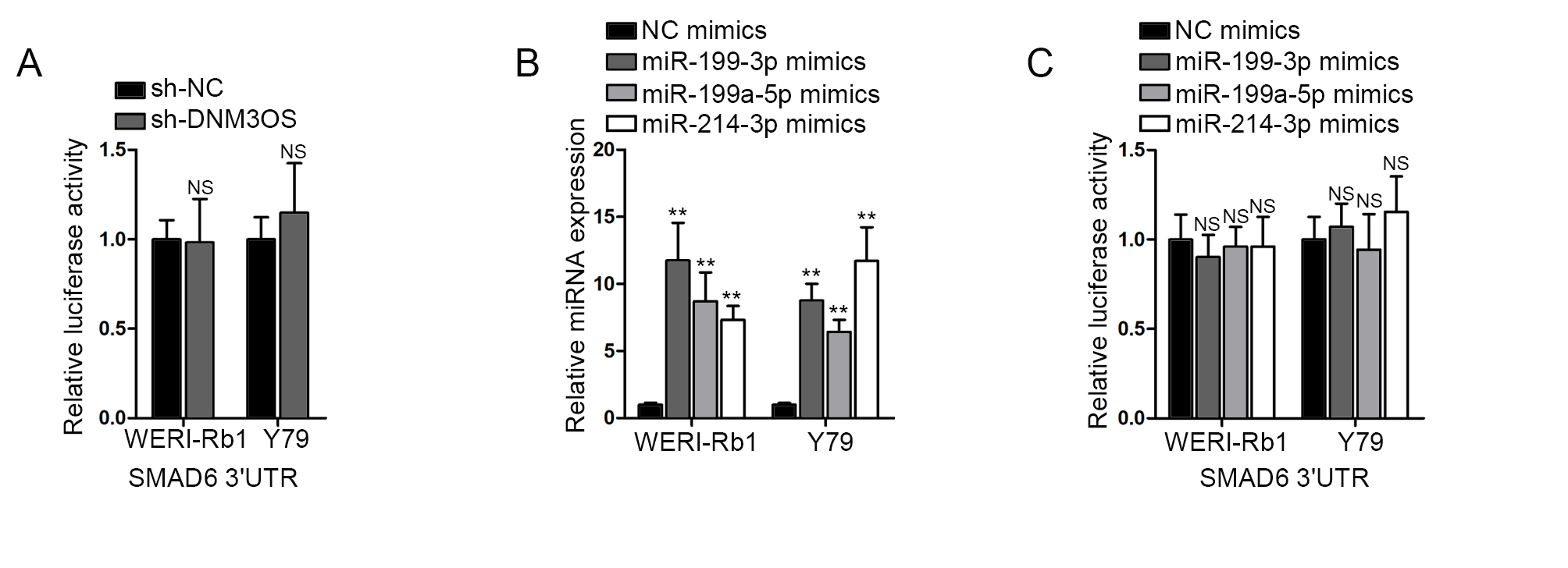

Supplement: Supplementary file 5 — Additional file 5: Figure S5. (A) Luciferase activity of vectors containing SMAD6 3′UTR was measured in RB cells in response to the silencing of DNM3OS. (B) Overexpression of miR-199-3p, miR-199a-5p and miR-214-3p in two RB cells by indicated miRNA mimics. (C) Luciferase activity of vectors containing SMAD6 3’UTR was measured in RB cells after inducing the upregulation of three miRNAs. **p < 0.01; NS: no significance. [file 12935_2020_1103_MOESM5_ESM.tif]
